# Real-world treatments and outcomes in metastatic and recurrent esophageal squamous cell carcinoma in Japan: a retrospective analysis using the Millennial Medical Record database

**DOI:** 10.1007/s10388-026-01233-z

**Published:** 2026-07-20

**Authors:** Ken Kato, Junice Ng, Tomoyoshi Nakano, Viriya Hantrakun, See-Hwee Yeo, Xiaowei Zhang, Hiroya Takeuchi

**Affiliations:** 1 Department of Head and Neck, Esophageal Medical Oncology, National Cancer Center Hospital, Tokyo, Japan; 2 Health Economics and Outcomes Research, BeOne Medicines, Ltd., Singapore, Singapore; 3 Medical Affairs, BeOne Medicines, Ltd., Tokyo, Japan; 4 Real World Solutions, IQVIA Solutions Asia, Bangkok, Thailand; 5 Real World Solutions, IQVIA Solutions Asia, Singapore, Singapore; 6 Statistics, BeOne Medicines, Ltd., Beijing, China; 7 Department of Surgery, Hamamatsu University School of Medicine, Hamamatsu, Shizuoka Japan

**Keywords:** Esophageal squamous cell carcinoma, Cisplatin, Immune checkpoint inhibitors, Chemotherapy regimens, Japan

## Abstract

**Background:**

Esophageal squamous cell carcinoma (ESCC) remains a significant clinical challenge in Japan. Real-world data on treatment patterns and outcomes, particularly for patients unfit for cisplatin, remain limited. This study evaluated characteristics, treatments, and outcomes of patients newly diagnosed with metastatic ESCC (mESCC) or recurrent ESCC (rESCC).

**Methods:**

A retrospective cohort analysis was conducted using the Millennial Medical Record database (December 2021–June 2024), including patients with m/rESCC. Patients receiving first-line (1 L) systemic therapy were categorized into cisplatin or non–cisplatin groups. Key outcomes included time-to-treatment discontinuation and overall survival (OS).

**Results:**

Among 155 patients, 52.3% had mESCC and 47.7% had rESCC; 31.0% received 1 L systemic therapy, 46.4% underwent surgery or radiotherapy, and 22.6% received no treatment. Among those receiving 1 L therapy, 58.3% were treated with immune checkpoint inhibitors (ICIs), either in combination with chemotherapy or alone. Non–cisplatin patients were older with greater comorbidity burden; ~33.3% receiving ICI monotherapy and 27.8% dual ICI therapy. In contrast, 56.7% of patients in the cisplatin group received a cisplatin–ICI combination. Treatment discontinuation was more frequent with cisplatin than non-cisplatin therapy (73.3% vs. 55.6%), despite longer median treatment duration (75 vs. 47 days). Twelve-month OS was higher with cisplatin than non‑cisplatin regimens (92.1% vs. 75.0%).

**Conclusions:**

In this real-world Japanese study, cisplatin-based regimens were commonly used for m/rESCC. Many patients who did not receive cisplatin had differing outcomes. This highlights the unmet need for better-tolerated alternatives for cisplatin-unfit patients with ESCC in Japan.

**Supplementary Information:**

The online version contains supplementary material available at https://doi.org/10.1007/s10388-026-01233-z.

## Introduction

Esophageal squamous cell carcinoma (ESCC) accounts for 90% of esophageal cancer cases in Japan, with approximately 18,000 diagnoses annually [1]. Despite advances in screening and treatment, ESCC is frequently diagnosed at an advanced stage and is associated with a poor prognosis. Recurrence remains common, often occurring within 8.3 months [2], and long-term survival remains limited, with a five-year overall survival (OS) rate of approximately 20% [3, 4]. First-line (1 L) therapy for metastatic or recurrent ESCC (m/rESCC) typically includes cisplatin and fluorouracil (5-FU), often combined with immune checkpoint inhibitors (ICIs) [5, 6]. However, cisplatin use is limited by toxicity and contraindications related to older age, renal impairment, neuropathy, and poor performance status [7, 8], prompting evaluation of alternatives.

Evidence indicates that cisplatin-unfit patients can achieve comparable outcomes using alternative platinum-based chemotherapy (CT) regimens such as FOLFOX (oxaliplatin + 5-FU/leucovorin) [9], CAPOX (capecitabine + oxaliplatin) [10], or carboplatin–5-FU [11], with manageable toxicity. Guidelines from the Japanese Esophageal Society (JES), ESMO, and NCCN recommend chemo-immunotherapy and nivolumab-ipilimumab dual immunotherapy (IO) as the preferred 1 L treatment for m/rESCC, regardless of cisplatin eligibility [7, 12, 13]. However, in Japan, cisplatin remains the primary 1 L platinum per guidelines, and oxaliplatin and carboplatin are less utilized [7]. ICIs targeting PD-1/PD-L1 have been approved for the treatment of advanced and recurrent ESCC in Japan since 2020 [14], following demonstrated efficacy in clinical trials [6, 15, 16]. However, real-world data on use of non-cisplatin regimens in combination with immunotherapy in for m/rESCC remain limited in Japan.

This study evaluated the characteristics, treatment patterns, and outcomes in Japanese patients with m/rESCC, comparing OS, time-to-treatment discontinuation (TTD), and time-to-next treatment (TTNT) between cisplatin and non-cisplatin 1 L regimens.

## Methods

### Study design and data source

This retrospective cohort study utilized de-identified patient-level data from the Millennial Medical Record (MMR) database in Japan [17, 18]. The MMR database integrates electronic health records, administrative claims, and hospital data from multiple healthcare institutions nationwide, enabling longitudinal tracking of clinical histories and treatment outcomes.

### Patient selection

Patients with histologically confirmed m/rESCC diagnosed between Dec 1, 2021 and Jun 30, 2023 were identified in MMR using ICD‑10 C15.x plus documented histology (Supplementary Table 1). Newly diagnosed mESCC was defined as first mESCC with no prior ESCC record in the preceding six months, rESCC was defined as the first documented recurrence after prior treatment, and patients meeting both criteria were classified as rESCC. The index date was the earliest date meeting mESCC or rESCC criteria, and patients were followed until Jun 30, 2024 to ensure a minimum follow-up period of 12 months for all patients.

### Data collection and definition

Baseline demographic and clinical characteristics (age, sex, smoking history, tumor location, metastatic status, Charlson Comorbidity Index [CCI], creatinine clearance [CrCl], and estimated glomerular filtration rate [eGFR]) were extracted from the database. 1 L systemic therapy was defined as CT and/or IO initiated on or after the index date, excluding neoadjuvant (≤ 28 days pre‑surgery), post‑surgical adjuvant (≤ 180 days post‑neoadjuvant completion), or curative‑intent chemoradiotherapy (≥ 25 radiotherapy sessions with concurrent CT). Among 1 L recipients, regimens were classified as cisplatin‑ or non‑cisplatin‑based, with concurrent surgery/radiotherapy (S/RT) recorded. Patients who did not receive 1 L systemic therapy were categorized as S/RT alone or no treatment.

Primary outcomes included time-to-event measures from initiation of 1 L systemic therapy. TTD was defined as the interval from 1 L therapy initiation to all-cause permanent cessation (e.g., completion, progression, toxicity, or death), with those without an event censored at death or at last observation date. TTNT was defined as the time from the initiation of 1 L therapy to the initiation of second-line (2 L) systemic therapy. Patients who had not started a 2 L regimen by the end of follow-up were censored at death or at last observation date.

OS was calculated from the index date (diagnosis of m/rESCC) to all-cause death, assessing survival across the overall cohort, including patients not receiving systemic therapy. Survivors were censored at the last observation date. All patients, including those not receiving systemic therapy, were included in the OS analysis for descriptive comparisons between treatment groups. Missing data are presented in Supplementary Table 2.

### Statistical analysis

All analyses were descriptive with no imputation for missing data. As all eligible patients were included (*N* = 155), no sample size calculation was performed. Due to small subgroup sizes and limited statistical power, no formal group comparisons were conducted.

Time-to-event outcomes (TTD, TTNT, OS) were analyzed using the Kaplan–Meier method, with medians and 95% confidence intervals (CIs) estimated. Fixed-time OS at 6 and 12 months was also reported. To assess potential bias related to variability in the time from diagnosis to treatment initiation, a sensitivity analysis was conducted that defined OS from 1 L systemic therapy initiation among treated patients. In line with MMR database privacy policies (Life Data Initiative Inc. and NTT DATA Japan Corporation), results involving ≤ 2 patients were masked, and approximate summaries were presented when applicable. Analyses were conducted using R version 4.4.0 (R Core Team, 2024).

## Results

### ESCC patients

A total of 155 patients with ESCC were included. The cohort was predominantly male (82.6%) and had a history of smoking (84.5%). Of these, 52.3% were newly diagnosed with mESCC, and 47.7% had rESCC (Table 1). Most tumors were thoracic (85.2%), with 11.0% cervical and 1.9% at the esophagogastric junction. Metastatic disease was present in 69.7% of patients, primarily in lymph nodes (85.2%), followed by lung (21.3%), liver (13.9%), bone (12.0%), and brain (2.8%). Median CCI was 4.0 (range 2.0–8.0), indicating moderate-to-severe comorbidity burden. Renal impairment was common, ~ 30.3% had eGFR < 60 mL/min/1.73 m² (Chronic Kidney Disease [CKD] Stage ≥ 3) and 45.8% had CrCl < 60 mL/min, though over half retained adequate renal function. Time from diagnosis to the 1 L initiation was 2 days (range 1–93 days).

**Table 1:** Baseline characteristics

	Overall (N = 155)	First-line systemic therapy			Surgery/Radiotherapy (n = 72)	No treatment (n = 35)
		Total (n = 48)	Cisplatin (n = 30)	Non-Cisplatin (n = 18)		
Demographics, n (%)						
Age group, years						
< 75	95 (61.3)	34 (70.8)	23 (76.7)	11 (61.1)	42 (58.3)	19 (54.3)
≥ 75	60 (38.7)	14 (29.2)	7 (23.3)	7 (38.9)	30 (41.7)	16 (45.7)
Male	128 (82.6)	40 (83.3)	24 (80.0)	≤ 16 (≤ 88.0)	58 (80.6)	30 (85.7)
Smoking history	131 (84.5)	42 (87.5)	24 (80.0)	18 (100)	62 (86.1)	27 (77.1)
Charlson Comorbidity Index, median (IQR)	4.0 (2.0–8.0)	5.0 (2.8–8.2)	4.0 (3.0–8.0)	8.0 (2.0–9.8)	3.0 (2.0–8.0)	6.0 (3.0–8.0)
Disease characteristics, n (%)						
Disease Group						
mESCC	81 (52.3)	38 (79.2)	25 (83.3)	13 (72.2)	22 (30.6)	21 (60.0)
rESCC	74 (47.7)	10 (20.8)	5 (16.7)	5 (27.8)	50 (69.4)	14 (40.0)
Tumor location ^a^						
Cervical	17 (11.0)	6 (12.5)	≤ 2 (-) ^d^	5 (27.8)	7 (9.7)	4 (11.4)
Thoracic	132 (85.2)	39 (81.3)	26 (86.7)	13 (72.2)	64 (88.9)	29 (82.9)
Esophagogastric junction	3 (1.9)	≤ 2 (-) ^d^	≤ 2 (-) ^d^	0	≤ 2 (-) ^d^	0
Unknown	5 (3.2)	≤ 2 (-) ^d^	≤ 2 (-) ^d^	0	≤ 2 (-) ^d^	3 (8.6)
Metastasis ^b^	108 (69.7)	45 (93.8)	27 (90.0)	18 (100)	33 (45.8)	30 (85.7)
Site of metastasis ^b,c^						
Liver	15 (13.9)	7 (15.6)	4 (14.8)	> 2 (-) ^d^	≤ 2 (-) ^d^	7 (23.3)
Lung	23 (21.3)	13 (28.9)	4 (14.8)	9 (50.0)	6 (18.2)	4 (13.3)
Bone	13 (12.0)	5 (11.1)	> 2 (-) ^d^	≤ 2 (-) ^d^	4 (12.1)	4 (13.3)
Brain	3 (2.8)	≤ 2 (-) ^d^	≤ 2 (-) ^d^	0	≤ 2 (-) ^d^	≤ 2 (-) ^d^
Lymph nodes	92 (85.2)	40 (88.9)	24 (88.9)	16 (88.9)	28 (84.8)	24 (80.0)
Others	≤ 2 (-) ^d^	0	0	0	0	≤ 2 (-) ^d^
Laboratory findings, n (%)						
Estimated creatinine clearance (mL/min) ^b^						
≥ 90	11 (7.1)	≤ 2 (-) ^d^	≤ 2 (-) ^d^	≤ 2 (-) ^d^	> 2 (-) ^d^	≤ 2 (-) ^d^
60—89	54 (34.8)	18 (37.5)	14 (46.7)	4 (22.2)	24 (33.3)	12 (34.3)
30—59	63 (40.6)	24 (50.0)	14 (46.7)	10 (55.6)	28 (38.9)	11 (31.4)
15—29	5 (3.2)	0	0	0	≤ 2 (-) ^d^	4 (11.4)
< 15	3 (1.9)	≤ 2 (-) ^d^	0	≤ 2 (-) ^d^	0	≤ 2 (-) ^d^
Estimated glomerular filtration rate (mL/min/1.73 m^2^) ^b^						
Stage 1—Normal or high (≥ 90)	19 (12.3)	7 (14.6)	7 (23.3)	0	> 2 (-) ^d^	6 (17.1)
Stage 2—Mildly decreased (60 – 89)	70 (45.2)	22 (45.8)	12 (40.0)	10 (55.6)	34 (47.2)	14 (40.0)
Stage 3a—Mildly to moderate decreased (45 – 59)	30 (19.4)	13 (27.1)	10 (33.3)	3 (16.7)	12 (16.7)	5 (14.3)
Stage 3b—Moderately to severely decreased (30 – 44)	11 (7.1)	≤ 2 (-) ^d^	0	≤ 2 (-) ^d^	7 (9.7)	≤ 2 (-) ^d^
Stage 4—Severely decreased (15 – 29)	4 (2.6)	0	0	0	≤ 2 (-) ^d^	3 (8.6)
Stage 5—Kidney failure (< 15)	≤ 2 (-) ^d^	≤ 2 (-) ^d^	0	≤ 2 (-) ^d^	0	≤ 2 (-) ^d^

IQR, interquartile range; mESCC, metastatic esophageal squamous cell carcinoma; rESCC, recurrent esophageal squamous cell carcinoma; %, percentage (based on number of patients with non-missing data)

^a^Patients may have multiple tumor locations; percentages can exceed 100%

^b^Variable includes missing data (Supplementary Table S1)

^c^Patients may have multiple metastatic sites; percentages can exceed 100%, calculated among patients with metastasis

^d^Counts ≤ 2 are masked (“–”) per institutional privacy policy; additional suppression applied to prevent back-calculation. Zero values are shown as reported

## Overall ESCC treatments

**Fig. 1:**
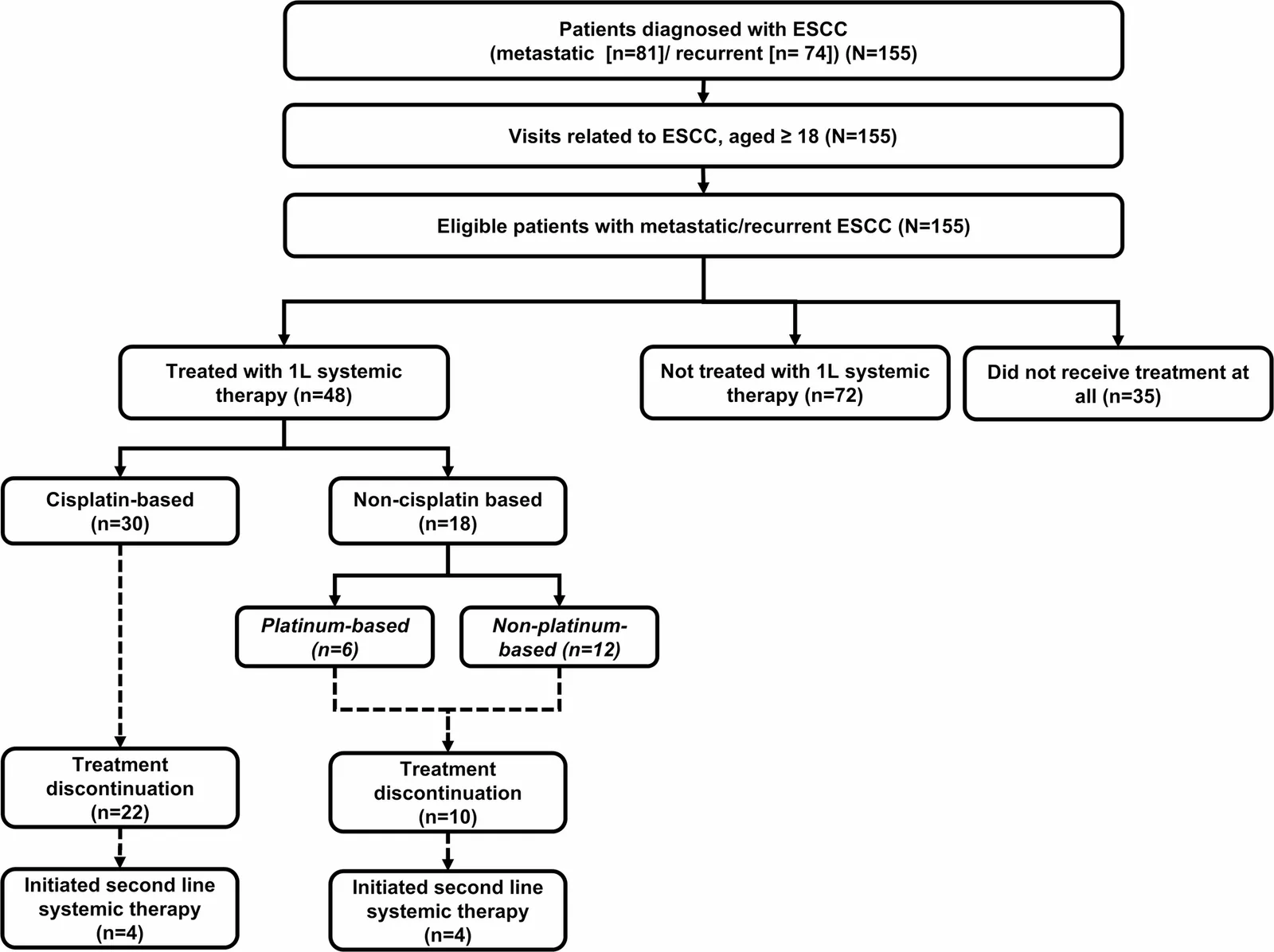
Study Flow

Among all patients, 48 (31.0%) received 1 L systemic therapy, 72 (46.4%) underwent S/RT, and 35 (22.6%) received no treatment (Fig. 1). The use of 1 L systemic therapy was more common in mESCC (46.9%) than rESCC (13.5%), with cisplatin regimens also more commonly used in mESCC (30.9% vs. 6.8%) (Supplementary Table 3). Compared to mESCC, patients with rESCC were more likely to receive S/RT (67.6% vs. 27.2%).

Compared with the S/RT group, patients receiving 1 L systemic therapy were younger (< 75 years: 70.8% vs. 58.3%) and more likely to present with distant metastases (93.8% vs. 45.8%) (Table 1). Thoracic ESCC was most prevalent in the S/RT group (88.9%), followed by no-treatment (82.9%) and 1 L therapy (81.3%) groups. CKD Stage ≥3a was more common in the 1 L group (~ 35.4% [≤ 17/48]) than S/RT (~ 29.1% [≤ 21/72]). Similarly, CrCl < 60 mL/min was observed in ~ 54.2% (≤ 26/48) of the 1 L group compared with ~ 41.7% (≤ 30/72) in S/RT group.

Within the 1 L cohort, the non‑cisplatin group were older (≥ 75 years: 38.9% vs. 23.3%) and had a higher comorbidity burden (median CCI: 8 vs. 4) (Table 1). Consistent with treatment selection, the IO‑only subgroup had the highest comorbidity burden (median CCI: 8) compared with CT‑only (median CCI: 4) and CT + IO (median CCI: 5) (Supplementary Table 4). CrCl < 60 mL/min was more frequent in the non–cisplatin group than in the cisplatin group (~ 66.7% vs. 46.7%), while renal impairment (eGFR < 60 mL/min/1.73 m^2^) was comparable between both groups (~ 38.9% vs. 33.3%). Among the S/RT group, 68.1% underwent surgical interventions (tumor resection or supportive procedures) and 34.7% received radiotherapy (Table 2).

**Table 2:** Treatments

	First-line systemic therapy			Surgery/Radiotherapy (n = 72)
	Total (n = 48)	Cisplatin (n = 30)	Non-Cisplatin (n = 18)	
Treatment at or after index date, n (%) ^b,c^				
Surgery	6 (12.5)	3 (10.0)	3 (16.7)	49 (68.1)
Tumor resection	≤ 2 (-) ^f^	≤ 2 (-) ^f^	0	37 (51.4)
Supportive surgery	5 (10.4)	≤ 2 (-) ^f^	3 (16.7)	15 (20.8)
Radiotherapy	12 (25.0)	8 (26.7)	4 (22.2)	25 (34.7)
Curative therapy ^d^	0	0	0	12 (16.7)
1L treatment ^e^	48 (100.0)	30 (100.0)	18 (100.0)	- ^a^
Chemotherapy only	20 (41.7)	13 (43.3)	> 2 (-) ^f^	- ^a^
Immunotherapy only	10 (20.8)	0	10 (55.6)	- ^a^
Chemotherapy and immunotherapy	18 (37.5)	17 (56.7)	≤ 2 (-) ^f^	- ^a^
1L therapy, n (%) ^c,e^				- ^a^
5-FU only	≤ 2 (-) ^f^	0	≤ 2 (-) ^f^	- ^a^
NDP only	≤ 2 (-) ^f^	0	≤ 2 (-) ^f^	- ^a^
NIVO only	4 (8.3)	0	4 (22.2)	- ^a^
PEMBRO only	≤ 2 (-) ^f^	0	≤ 2 (-) ^f^	- ^a^
S-1	≤ 2 (-) ^f^	0	≤ 2 (-) ^f^	- ^a^
CDDP + PEMBRO	≤ 2 (-) ^f^	≤ 2 (-) ^f^	0	- ^a^
5-FU + CDDP	8 (16.7)	8 (26.7)	0	- ^a^
5-FU + NDP	≤ 2 (-) ^f^	0	≤ 2 (-) ^f^	- ^a^
Ipilimumab + NIVO	5 (10.4)	0	5 (27.8)	- ^a^
5-FU + CDDP + Docetaxel	5 (10.4)	5 (16.7)	0	- ^a^
5-FU + CDDP + NIVO	5 (10.4)	5 (16.7)	0	- ^a^
5-FU + CDDP + PEMBRO	11 (22.9)	11 (36.7)	0	- ^a^
5-FU + NDP + PEMBRO	≤ 2 (-) ^f^	0	≤ 2 (-) ^f^	- ^a^
5-FU + Paclitaxel + NDP	≤ 2 (-) ^f^	0	≤ 2 (-) ^f^	- ^a^
5-FU + Levofolinate + Oxaliplatin + S-1	≤ 2 (-) ^f^	0	≤ 2 (-) ^f^	- ^a^
Duration of 1L therapy (days) ^e^				
Mean (SD)	40.8 (43.2)	50.9 (49.1)	24.3 (24.6)	- ^a^
Median (IQR)	29.0 (5.0–56.0)	42.0 (5.0–75.0)	18.5 (1.8–37.2)	- ^a^
Min; Max	1.0; 165.0	1.0; 165.0	1.0; 82.0	- ^a^

1 L, first-line therapy; 5-FU, fluorouracil; CDDP, cisplatin; ESCC, esophageal squamous cell carcinoma; NDP, nedaplatin; NIVO, nivolumab; PEMBRO, pembrolizumab; S-1, Tegafur + Gimeracil + Oteracil

^a^Not applicable; these patients did not receive systemic therapy

^b^Percentages may exceed 100% as patients could receive multiple treatments

^c^Percentages calculated by column total

^d^Curative radiotherapy: chemotherapy and radiotherapy started within 2 days and ≥ 25 sessions

^e^1 L therapy includes chemotherapy and/or immunotherapy, excluding adjuvant/neoadjuvant therapy or therapy combined with curative radiotherapy

^f^Counts ≤ 2 are masked (“–”) per institutional privacy policy; suppression applied to prevent back-calculation. Zero values are shown as reported

### First-line systemic treatment

For patients receiving 1 L systemic treatment, CT accounted for 41.7% of the cases, followed by CT-IO combination at 37.5% and IO monotherapy at 20.8% (Table 2). In the cisplatin group, 56.7% received CT-IO and 43.3% received CT alone (Table 2). In contrast, IO (monotherapy or dual-agent regimens) was the most frequently prescribed approach in the non–cisplatin group (~ 55.6%). Alternative platinum agents in the non-cisplatin group included nedaplatin (≤ 8/18, ~ 44.4%) and oxaliplatin (≤ 2/18, ~ 11.1%). Additionally, S-1 (tegafur + gimeracil + oteracil), with or without platinum agents, was used in ≤ 4/18 cases (~ 22.2%). Overall, non-cisplatin regimens were more diverse and had shorter median treatment duration compared to cisplatin regimens (18.5 vs. 42.0 days).

### Time-to-treatment discontinuation and time-to-next treatment

**Fig. 2:**
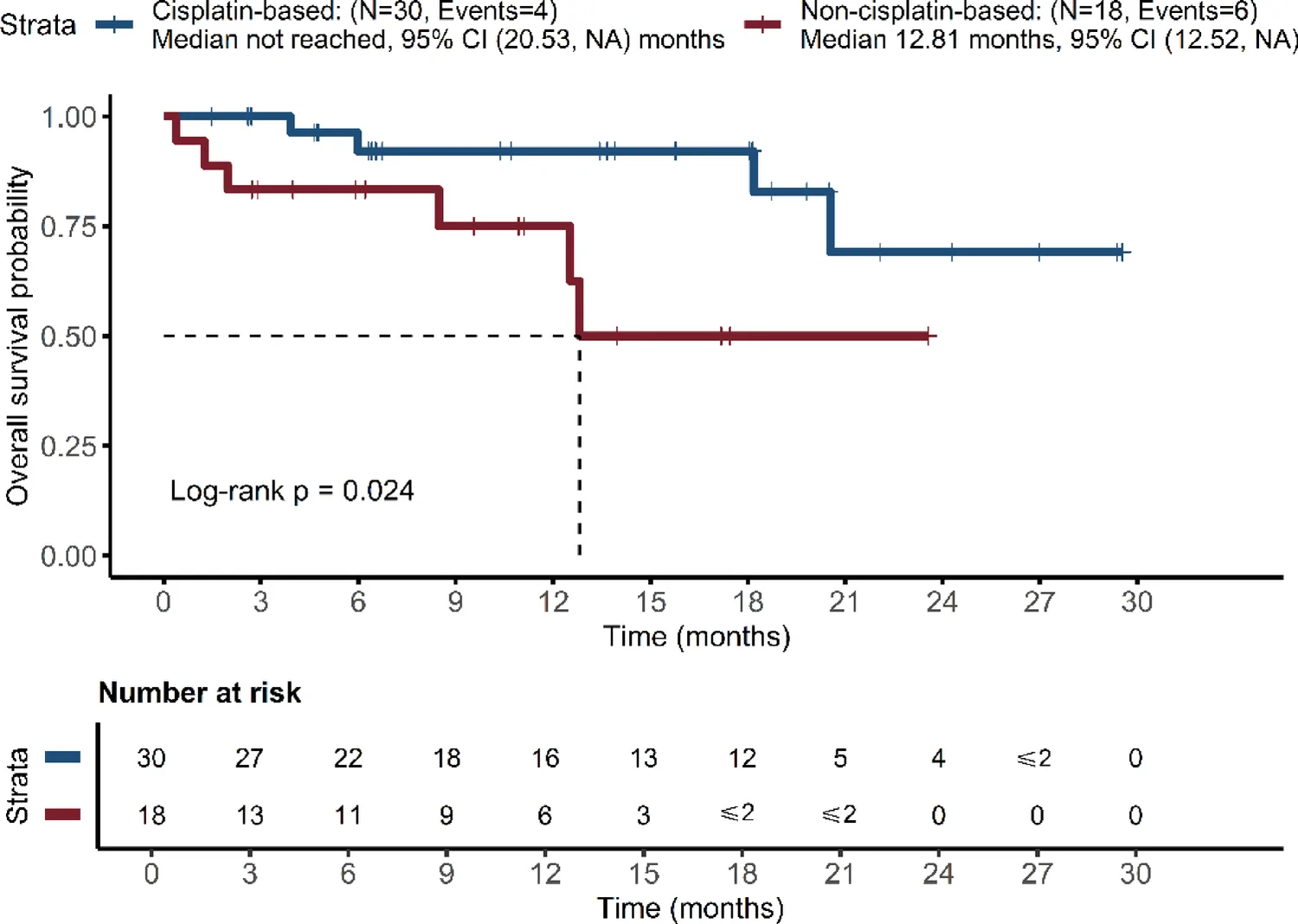
Kaplan–Meier curve for time to treatment discontinuation by first-line regimen. Counts ≤ 2 are masked per institutional privacy policy. Zero values (0) and missing data are shown as reported

Among patients receiving 1 L systemic treatment, 66.7% (32/48) discontinued treatment, with a higher rate of discontinuation in the cisplatin group than the non-cisplatin group (73.3% vs. 55.6%; Table 3). However, the median TTD was longer in the cisplatin group (75 days) than in the non-cisplatin group (47 days) (Fig. 2; Table 3). Although discontinuation rate was high, overall, only eight patients initiated 2 L therapy (16.7%), with four patients in each group (cisplatin 13.3%, non-cisplatin 22.2%; Table 3). The median TTNT was not estimable as most patients in the 1 L group (75.0% [24/32]) did not receive subsequent systemic therapy within the observation period.

**Table 3:** Time-to-event outcomes

	First-line systemic therapy			Surgery/Radiotherapy	No treatment
	Total	Cisplatin	Non-Cisplatin		
Time-to-treatment discontinuation, n (%)					
At-risk patients	48	30	18	- ^g^	- ^g^
Cumulative therapy discontinuations	32 (66.7)	22 (73.3)	10 (55.6)	- ^g^	- ^g^
Cumulative censored patients	16 (33.3)	8 (26.7)	8 (44.4)	- ^g^	- ^g^
Alive	0	0	0	- ^g^	- ^g^
Deceased	5 (10.4)	≤ 2 (-) ^h^	> 2 (-) ^h^	- ^g^	- ^g^
Lost to follow-up	11 (22.9)	> 2 (-) ^h^	> 2 (-) ^h^	- ^g^	- ^g^
Median follow-up (95% CI), days ^a^	143 (116–415)	181 (140–NE) ^f^	97 (59–179)	- ^g^	- ^g^
Median time to discontinuation (95% CI), days ^b^	56.0 (42.0–145.0)	75.0 (47.0–165.0)	47.0 (15.0–NE)^f^	- ^g^	- ^g^
Time-to-next-treatment, n (%)				- ^g^	- ^g^
At-risk patients	48	30	18	- ^g^	- ^g^
Cumulative second-line therapy initiations	8 (16.7)	4 (13.3)	4 (22.2)	- ^g^	- ^g^
Cumulative censored patients	40 (83.3)	26 (86.7)	14 (77.8)	- ^g^	- ^g^
Alive	24 (50.0)	18 (60.0)	6 (33.3)	- ^g^	- ^g^
Deceased	5 (10.4)	≤ 2 (-) ^h^	3 (16.7)	- ^g^	- ^g^
Lost to follow-up	11 (22.9)	> 2 (-) ^h^	5 (27.8)	- ^g^	- ^g^
Median follow-up time (95% CI), days ^a^	274 (140–404)	274 (139–414)	285 (97–369)	- ^g^	- ^g^
Median time to next treatment (95% CI), days ^b^	NE ^f^	NE ^f^	NE (288.0–NE) ^f^	- ^g^	- ^g^
Overall survival, n (%)					
At-risk patients	48	30	18	72	35
Cumulative deaths ^c^	10 (20.8)	4 (13.3)	6 (33.3)	4 (5.6)	10 (28.6)
Cumulative censored patients ^c^	38 (79.2)	26 (86.7)	12 (66.7)	68 (94.4)	25 (71.4)
Alive	26 (54.2)	19 (63.3)	7 (38.9)	34 (47.2)	13 (37.1)
Lost to follow-up	12 (25.0)	7 (23.3)	5 (27.8)	34 (47.2)	12 (34.3)
Median follow-up (95% CI), days ^d^	416 (291–531)	423 (205–570)	338 (189–523)	333 (282–472)	217 (128–367)
Overall survival rate, % (95% CI)^e^					
6 months	91.4 (78.6–96.7)	96.3 (76.5–99.5)	83.3 (56.8–94.3)	95.8 (87.4–98.6)	69.0 (49.3–82.4)
12 months	85.5 (70.1–93.3)	92.1 (71.9–98.0)	75.0 (45.2–90.1)	93.8 (84.0–97.6)	69.0 (49.3–82.4)

CI, confidence interval; NE, not estimable

^a^Reverse KM estimate among patients initiating 1 L systemic therapy

^b^Estimated using KM method among patients who initiated 1 L therapy

^c^Percentages based on patients at risk

^d^Reverse KM estimate

^e^95% CI calculated using log–log transformation

^f^Median and/or CI not estimable due to insufficient events (< 50%) or heavy censoring

^g^Not applicable; these patients did not receive 1 L therapy

^h^Counts ≤ 2 are masked (“–”) per institutional privacy policy; suppression applied to prevent back-calculation. Zero values are shown as reported

### Real-world overall survival

**Fig. 3:**
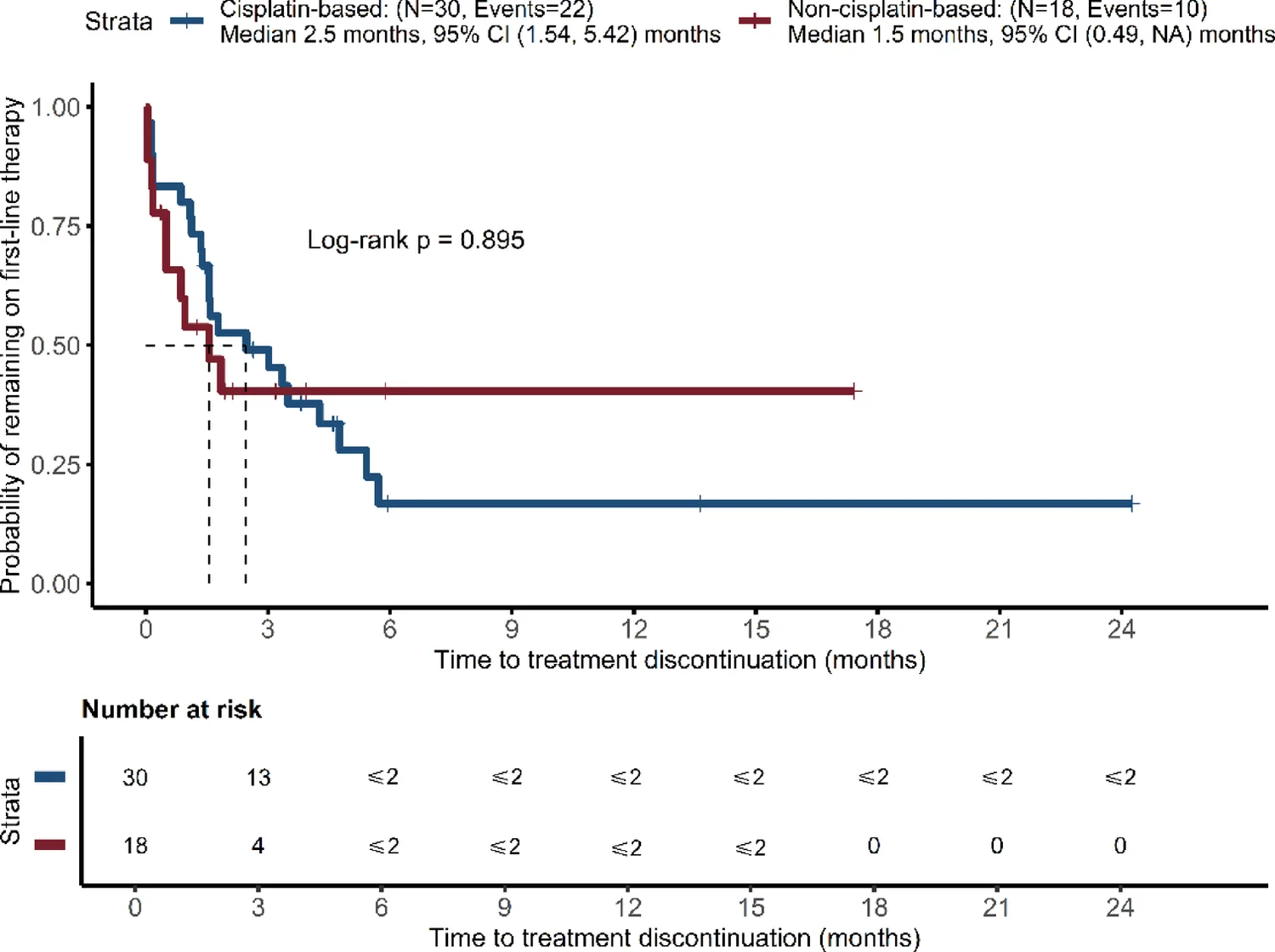
Kaplan-Meier curve for overall survival by first-line regimen. Counts ≤ 2 are masked per institutional privacy policy. Zero values (0) and missing data are shown as reported

Among patients receiving 1 L systemic therapy, the median follow‑up for OS was 416 days (95% CI, 291–531), with longer follow-up in the cisplatin group (423 days) than the non-cisplatin group (338 days). By the end of follow‑up, 20.8% of patients in the 1 L cohort had died. The cumulative mortality was 13.3% in the cisplatin group and 33.3% in the non‑cisplatin group. The 12‑month OS was 92.1% for the cisplatin group and 75.0% for the non-cisplatin group. The 6‑month OS rate was 96.3% and 83.3%, respectively. Sensitivity analyses measuring OS from 1 L initiation showed similar results (Supplementary Table 5). Among 1 L‑treated patients, the 12‑month OS rate was 85.5% overall and 84.4%, 72.0%, and 94.1% in those receiving CT alone, IO alone, and CT + IO, respectively (Supplementary Table 6). Median OS was not reached in the cisplatin subgroup, while estimated median OS for the non‑cisplatin subgroup was 12.8 months (Fig. 3).

For patients managed without 1 L therapy, the median follow-up time for OS was 333 days (95% CI, 282–472) in the S/RT group and 217 days (95% CI, 128–367) in the no-treatment group (Table 3). By the end of the follow-up, 5.6% of patients in the S/RT group and 28.6% in the no-anticancer treatment group had died.

## Discussion

This retrospective analysis provides real-world insights into treatment patterns and outcomes among patients with mESCC and rESCC during the early phase of IO adoption in Japan, highlighting several unmet needs for this population.

Our study showed that cisplatin-based doublet CT remains the standard 1 L therapy, consistent with Japanese and global practice [7]. Observed 12-month survival was higher with cisplatin-containing regimens (with or without IO) than non-cisplatin regimens; however, without statistical comparisons and adjustment, these findings are descriptive and likely reflect confounding by indication rather than a true treatment effect. Notably, uptake of 1 L systemic therapy was low, with few progressing to 2 L. As this study included only non-specialist hospitals, the findings likely reflect care in these settings, where patients are older and have greater disease burden and comorbidity, limiting intensive 1 L therapy.

The non–cisplatin cohort was heterogeneous, including IO-only treatments (single-agent or dual ICI) and alternative platinum-based regimens (e.g., nedaplatin or oxaliplatin). Patients in this group were generally older and had more comorbidities, which may have contributed to shorter treatment duration and lower survival. Many non–cisplatin patients discontinued therapy early; often after fewer cycles than the typical 4–6 in 1 L ESCC treatment [15], suggesting poor tolerance or rapid disease progression in this frail population, consistent with prior reports in older, “trial‑ineligible” ESCC patients [19]. These findings highlight the need to better understand drivers of discontinuation and factors in selection of non‑cisplatin regimens, including clinical ineligibility for cisplatin.

In Japan, JES guidelines recommend FOLFOX as a 1 L option for patients unfit for cisplatin (e.g., age ≥ 75 years, renal or cardiac dysfunction, poor performance status) and endorse nedaplatin for those with renal or cardiac dysfunction [7, 20]. Other oxaliplatin‑containing regimens (e.g., CAPOX) are not included in this guideline. In our cohort, however, FOLFOX was not used and oxaliplatin‑based therapy was infrequently prescribed; non–cisplatin regimens were primarily IO‑based treatments, with a few patients receiving non‑standard combinations of 5‑FU (± levofolinate) plus oxaliplatin and S‑1. This pattern likely reflects institutional preferences, clinician familiarity with cisplatin‑based therapy, cautious selection for frail patients, and practical factors such as regulatory timing, reimbursement, and formulary availability. Together, these findings suggest a gap between guideline recommendations and real‑world practice. Meta‑analyses have shown these doublets to be effective and tolerable compared with other platinum regimens [21], and international guidelines recognize them as alternatives for patients unsuitable for cisplatin [13]. These data support the potential value of oxaliplatin-containing therapy for patients unable to tolerate cisplatin or intensive therapy.

Despite having traditional high‑risk features (advanced age or renal impairment), some patients still received cisplatin, indicating that these factors alone did not preclude its use. In routine practice, such clinical considerations are assessed in combination rather than as rigid thresholds, with 1 L treatment decisions made on a case‑by‑case basis, integrating multiple clinical considerations, including comorbidities affecting hydration tolerance (e.g., cardiac dysfunction), overall frailty, cognitive status, and prior cisplatin exposure. This highlights the complexity of real‑world decision‑making and the need for clearer, clinically applicable definitions of cisplatin eligibility.

Our study found that a large proportion of patients did not receive systemic therapy, consistent with real‑world reports in which treatment is often limited by comorbidities and poor functional status [10, 19]. Future studies should identify predictors of treatment tolerance, evaluate less‑intensive regimens, and incorporate geriatric assessments to better guide individualized therapy [22–24]. Expanding access to tolerable treatment options may improve outcomes for this underserved population.

This study has several limitations inherent to its retrospective, real-world design. Given the observational nature of the analysis and the incomplete availability of key prognostic factors (e.g., ECOG performance status and PD‑L1 expression), confounding by indication cannot be ruled out; thus, observed outcomes differences should be interpreted with caution. Although proxies for patient fitness (age, comorbidities, renal function) were assessed, they do not fully capture clinical status, precluding formal reclassification of cisplatin eligibility. The small sample size, especially in the non-cisplatin subgroup, limited statistical power and generalizability, and exploratory analyses among potentially cisplatin-unfit patients (ECOG ≥ 1, heart failure, eGFR < 60 mL/min/1.73 m²) were inconclusive. ICI uptake in our cohort may have been lower than in current practice, as the study period partially overlapped with the introduction of pembrolizumab and nivolumab in Japan. Lastly, as data were not drawn from cancer-specialty centers, findings may best represent university and regional hospitals in Japan.

Despite these limitations, this study provides insight into real‑world management and outcomes of m/rESCC in Japan. The findings reaffirm the role of cisplatin‑based regimens for patients who can tolerate them, while highlighting the complexity of treatment decisions for those unable to receive cisplatin and the need for clearer, clinically applicable eligibility definitions and evidence‑based use of alternative regimens.

## Conclusions

This real‑world analysis shows that cisplatin‑based regimens remain commonly used in routine practice for patients with m/rESCC in Japan. The differing outcomes observed with non‑cisplatin regimens, along with the marked variability in care among cisplatin‑unfit patients highlight the need for clinically applicable eligibility definitions and local evidence for integrating alternative regimens, including oxaliplatin- or nedaplatin-based therapies and combinations with ICIs.

## Supplementary Information

Below is the link to the electronic supplementary material.

Supplementary Material 1

## Data Availability

In accordance with the data privacy policies of MMR, managed by Life Data Initiative Inc. and NTT DATA Japan Corporation, this study was not approved for external data sharing. Therefore, the dataset is not available beyond what is included in the manuscript. Any future secondary use of the data will require separate approval from the relevant data governance authorities.
