# Supplementary material for: Real-world treatments and outcomes in metastatic and recurrent esophageal squamous cell carcinoma in Japan: a retrospective analysis using the Millennial Medical Record database
Source: Esophagus. 2026 Jul 20;23(4):829–38. doi: 10.1007/s10388-026-01233-z (PMC13723668; doi:10.1007/s10388-026-01233-z)
Supplement: Supplementary file 1 [file 10388_2026_1233_MOESM1_ESM.docx]

# Real-World Treatments and Outcomes in Metastatic and Recurrent Esophageal Squamous Cell Carcinoma in Japan: A Retrospective Analysis Using the Millennial Medical Record Database

Ken Kato^1^, Junice Ng^2^, Tomoyoshi Nakano^3^, Viriya Hantrakun^4^, See-Hwee Yeo^5^, Xiaowei Zhang^6^, Hiroya Takeuchi^7^

^1^ Department of Head and Neck, Esophageal Medical Oncology, National Cancer Center Hospital, Tokyo, Japan.

^2^ Health Economics and Outcomes Research, BeOne Medicines, Ltd., Singapore

^3^ Medical Affairs, BeOne Medicines, Ltd., Japan

^4^ Real World Solutions, IQVIA Solutions Asia, Bangkok, Thailand

^5^ Real World Solutions, IQVIA Solutions Asia, Singapore, Singapore

^6^ Statistics, BeOne Medicines, Ltd., China

^7^ Department of Surgery, Hamamatsu University School of Medicine, Hamamatsu, Shizuoka, Japan

Corresponding author: Dr Ken Kato

Department of Head and Neck, Esophageal Medical Oncology, National Cancer Center Hospital, Tokyo, Japan

Email: [kenkato@ncc.go.jp](mailto:kenkato@ncc.go.jp)

# Supplementary Table 1 Eligibility Criteria

### **Inclusion criteria**

For mESCC patients:

Newly diagnosed with and histopathologically confirmed to have stage IV mESCC between December 01, 2021, and June 30, 2024, and verified using the 6-month baseline period (i.e. no previous record of mESCC diagnosis before index date).

- Identified using the DPC and International Classification of Diseases, Tenth Revision (ICD-10) codes (i.e., ‘C15.X’ representing malignant neoplasm of esophagus), AND
- Documented histology of EC as squamous cell carcinoma, AND
- Documented diagnosis as stage IV ESCC, OR with the TNM criteria^a^:
  - Clinical stage: T4, N0-3 and M1 or T0-4, N0-3 and M1b
  - Pathological stage: T4b, N0-3 and M1 or T4a, N2-3 and M1 or T3, N3 and M1 or T0-4, N0-3 and M1b

*^a^Histopathological confirmation was conducted by the MMR team prior to sharing the study data with the research team. As a result, TNM staging information was not available in this study.*

For first recurrent ESCC patients:

Histopathologically confirmed first ESCC recurrence (stages I-IV) between December 01, 2021, and June 30, 2024, and verified using all records available in MMR (i.e., no previous record of ESCC recurrence before index date).

- Identified using the DPC and ICD-10 codes (i.e., C15.X’ representing malignant neoplasm of esophagus), AND
- Documented histology of EC as squamous cell carcinoma, AND
- Documented clinical status of ESCC recurrence.

### **Exclusion Criteria**

None.

# Supplementary Table 2 Summary of missing values for baseline characteristics

|  |  | **First-line systemic therapy** | | | **Surgery/ Radiotherapy (n=72)** | **No treatment  (n=35)** |
| --- | --- | --- | --- | --- | --- | --- |
|  | **Overall**  **(N=155)** | **Total (N=48)** | **Cisplatin (n=30)** | **Non-Cisplatin (n=18)** |  |  |
| Metastasis, n (%) | 13 (8.4) | 0 | 0 | 0 | 11 (15.2) | ≤2 (-) ^a^ |
| Estimated creatinine clearance, CrCl (mL/min) ^b^ | 19 (12.3) | 3 (6.3) | ≤2 (-) ^a^ | ≤2 (-) ^a^ | 12 (16.7) | 4 (11.4) |
| Estimated glomerular filtration rate, eGFR (mL/min/1.73m²) ^b^ | 19 (12.3) | 3 (6.3) | ≤2 (-) ^a^ | ≤2 (-) ^a^ | 12 (16.7) | 4 (11.4) |

^a^ Counts ≤2 are masked (“–”) in accordance with institutional data privacy and patient confidentiality policy. Additional suppression is applied where necessary to prevent back-calculation. Zero values are shown as reported

# Supplementary Table 3 Patient disposition

|  | **Eligible patients, n (%) (N=155)** | | |
| --- | --- | --- | --- |
|  | Overall | With metastatic ESCC (n=81 [52.3%]) | With recurrent ESCC  (n=74 [47.7%]) |
| **Treated with 1L systemic therapy** | **48 (31.0)** | **38 (46.9)** | **10 (13.5)** |
| Cisplatin treatment | 30 (19.3) | 25 (30.9) | 5 (6.8) |
| Non-cisplatin treatment | 18 (11.6) | 13 (16.0) | 5 (6.8) |
| - Platinum-based | 6 (3.9) | 3 (3.7) | >2 (-) ^a^ |
| - Non-platinum-based | 12 (7.7) | 10 (12.3) | ≤2 (-) ^a^ |
| **Surgery/ Radiotherapy** | **72 (46.4)** | **22 (27.2)** | **50 (67.6)** |
| Surgery ^b^ | 49 (31.6) | 11 (13.6) | 38 (51.4) |
| Radiotherapy ^b^ | 25 (16.1) | 13 (16.0) | 12 (16.2) |
| **No treatment** | **35 (22.6)** | **21 (25.9)** | **14 (18.9)** |

Abbreviation: ESCC, esophageal squamous cell carcinoma.

Percentages were calculated by column total.

^a^ Counts ≤2 are masked (“–”) in accordance with institutional data privacy and patient confidentiality policy.

^b^ Surgery and radiotherapy are not mutually exclusive; patients could receive one or both treatments.

# Supplementary Table 4 Baseline characteristics by type of treatment among those receiving first-line systemic therapy

|  | **Total (N=48)** | **CT only (n=20)** | **IO only (n=10)** | **CT + IO (n=18)** |
| --- | --- | --- | --- | --- |
| **Demographics, n (%)** |  |  |  |  |
| Age group in years |  |  |  |  |
| <75 | 34 (70.8) | 12 (60.0) | >2 (-) ^c^ | >2 (-) ^c^ |
| ≥75 | 14 (29.2) | 8 (40.0) | ≤2 (-) ^c^ | >2 (-) ^c^ |
| Male | 40 (83.3) | 18 (90.0) | 8 (80.0) | 14 (77.8) |
| Smoking history | 42 (87.5) | 18 (90.0) | 10 (100) | 14 (77.8) |
| Charlson Comorbidity Index, median (IQR) | 5.0 (2.8–8.2) | 4.0 (3.0–8.0) | 8.0 (2.0–9.0) | 5.0 (3.0–8.0) |
| **Disease characteristics, n (%)** |  |  |  |  |
| **Disease Group** |  |  |  |  |
| mESCC | 38 (79.2) | 16 (80.0) | >2 (-) ^c^ | >2 (-) ^c^ |
| rESCC | 10 (20.8) | 4 (20.0) | ≤2 (-) ^c^ | >2 (-) ^c^ |
| **Tumor location ^a^** |  |  |  |  |
| Cervical | 6 (12.5) | 3 (15.0) | ≤2 ^c^ | ≤2 (-) ^c^ |
| Thoracic | 39 (81.3) | 15 (75.0) | 8 (80.0) | 16 (88.9) |
| Esophagogastric junction | ≤2 (-) ^c^ | ≤2 (-) ^c^ | 0 | ≤2 (-) ^c^ |
| Unknown | ≤2 (-) ^c^ | ≤2 (-) ^c^ | 0 | 0 |
| **Metastasis** | 45 (93.8) | 18 (90.0) | 10 (100) | 17 (94.4) |
| **Site of metastasis ^b^** |  |  |  |  |
| Liver | 7 (15.6) |  | ≤2 (-) ^c^ | 4 (23.5) |
| Lung | 13 (28.9) | 6 (33.3) | 5 (50.0) | ≤2 (-) ^c^ |
| Bone | 5 (11.1) | 0 | ≤2 (-) ^c^ | 3 (17.6) |
| Brain | ≤2 (-) ^c^ | 0 | 0 | ≤2 (-) ^c^ |
| Lymph nodes | 40 (88.9) | 18 (100) | 8 (80.0) | 14 (82.4) |
| **Laboratory findings, n (%)** |  |  |  |  |
| Estimated creatinine clearance, CrCl (mL/min) |  |  |  |  |
| ≥90 | ≤2 (-) ^c^ | ≤2 (-) ^c^ | 0 | ≤2 (-) ^c^ |
| 60 - 89 | 18 (40.0) | 5 (29.4) | 3 (30.0) | 10 (55.6) |
| 30 - 59 | 24 (53.3) | 11 (64.7) | 6 (60.0) | 7 (38.9) |
| 15 - 29 | 0 | 0 | 0 | 0 |
| <15 | ≤2 (-) ^c^ | 0 | ≤2 (-) ^c^ | 0 |
| Missing | 3 | 3 | 0 | 0 |
| **Estimated glomerular filtration rate, eGFR (mL/min/1.73m²)** |  |  |  |  |
| Stage 1 (Normal or high): ≥90 | 7 (15.6) | 3 (17.6) | 0 | 4 (22.2) |
| Stage 2 (Mildly decreased): 60 – 89 | 22 (48.9) | 7 (41.2) | 6 (60.0) | 9 (50.0) |
| Stage 3a (Mildly to moderate decreased): 45 – 59 | 13 (28.9) | 6 (35.3) | ≤2 (-) ^c^ | 5 (27.8) |
| Stage 3b (Moderately to severely decreased): 30 – 44 | ≤2 (-) ^c^ | ≤2 (-) ^c^ | ≤2 (-) ^c^ | 0 |
| Stage 4 (Severely decreased): 15 – 29 | 0 | 0 | 0 | 0 |
| Stage 5 (Kidney failure): <15 | ≤2 (-) ^c^ | 0 | ≤2 (-) ^c^ | 0 |
| Missing | 3 | 3 | 0 | 0 |

Abbreviations: CT, chemotherapy; IO, immunotherapy; IQR, interquartile range; mESCC, metastatic esophageal squamous cell carcinoma; rESCC, recurrent esophageal squamous cell carcinoma; %, percentage (based on number of patients with non-missing data).

^a^ Patients may have multiple tumor locations; percentages can exceed 100%.

^b^ Patients may have multiple metastatic sites; percentages can exceed 100%, calculated among patients with metastasis.

^c^ Counts ≤2 are masked (“–”) per institutional privacy policy; additional suppression applied to prevent back-calculation. Zero values are shown as reported

# Supplementary Table 5 Real-world overall survival from first-line therapy initiation among 1L systemic therapy recipients

|  | **Overall** | **Cisplatin** | **Non-Cisplatin** |
| --- | --- | --- | --- |
| **At-risk patients** | 48 | 30 | 18 |
| **Status at end of follow-up** |  |  |  |
| Cumulative deaths, n (%) ^a^ | 10 (20.8) | 4 (13.3) | 6 (33.3) |
| Cumulative censored patients, n (%) ^a^ | 38 (79.2) | 26 (86.7) | 12 (66.7) |
| Alive | 26 (54.2) | 19 (63.3) | 7 (38.9) |
| Lost to follow up | 12 (25.0) | 7 (23.3) | 5 (27.8) |
| **Overall survival from first-line therapy initiation** |  |  |  |
| Median follow-up time (95% CI) (days) ^b^ | 415 (290, 522) | 423 (204, 550) | 337 (179, 522) |
| Overall survival rate, % (95% CI) ^c^ |  |  |  |
| 6 months | 91.3 (78.4, 96.7) | 96.3 (76.5, 99.5) | 83.3 (56.8, 94.3) |
| 12 months | 85.4 (69.9, 93.3) | 92.1 (71.9, 98.0) | 75.0 (45.2, 90.1) |
| **Time to first-line therapy initiation, days** |  |  |  |
| Mean (SD) | 9.8 (18.5) | 10.5 (16.6) | 8.5 (21.8) |
| Median (Q1, Q3) | 2.0 (2.0, 3.2) | 2.0 (2.0, 4.0) | 2.0 (2.0, 2.0) |
| Min; Max | 1.0; 93.0 | 1.0; 50.0 | 1.0; 93.0 |

Abbreviations: CI, confidence interval; Q, quartile; SD, standard deviation

^a^ Percentages based on patients at-risk.

^b^ Reverse Kaplan-Meier method

^c^ 95% CI calculated using the log-log transformation.

# Supplementary Table 6 Real-world overall survival by type of treatment among 1L systemic therapy recipients

|  | **Total** | **CT only** | **IO only** | **CT + IO** |
| --- | --- | --- | --- | --- |
| **At-risk patients** | 48 | 20 | 10 | 18 |
| **Status at end of follow-up** |  |  |  |  |
| Cumulative deaths, n (%) ^a^ | 10 (20.8) | 5 (25.0) | 4 (40.0) | ≤2 (-) ^e^ |
| Cumulative censored patients, n (%) ^a^ | 38 (79.2) | 15 (75.0) | 6 (60.0) | >2 (-) ^e^ |
| Alive | 26 (54.2) | 12 (60.0) | ≤2 (-) ^e^ | 12 (66.7) |
| Lost to follow up | 12 (25.0) | 3 (15.0) | >2 (-) ^e^ | >2 (-) ^e^ |
| **Overall survival from index date** |  |  |  |  |
| Median follow-up time (95% CI) (days) ^b^ | 416 (291, 531) | 549 (338, 672) | 291 (180, NE) ^d^ | 316 (145, 423) |
| Overall survival rate, % (95% CI) ^c^ |  |  |  |  |
| 6 months | 91.4 (78.6, 96.7) | 90.0 (65.6, 97.4) | 90.0 (47.3, 98.5) | 93.8 (63.2, 99.1) |
| 12 months | 85.5 (70.1, 93.3) | 84.4 (58.9, 94.7) | 72.0 (23.8, 92.8) | 93.8 (63.2, 99.1) |
| **Overall survival from first-line therapy initiation** |  |  |  |  |
| Median follow-up time (95% CI) (days) ^b^ | 415 (290, 522) | 530 (337, 671) | 290 (120, NE) ^d^ | 315 (144, 423) |
| Overall survival rate, % (95% CI) ^c^ |  |  |  |  |
| 6 months | 91.3 (78.4, 96.7) | 90.0 (65.6, 97.4) | 90.0 (47.3, 98.5) | 93.8 (63.2, 99.1) |
| 12 months | 85.4 (69.9, 93.3) | 84.4 (58.9, 94.7) | 72.0 (23.8, 92.8) | 93.8 (63.2, 99.1) |
| **Time to first-line therapy initiation, days** |  |  |  |  |
| Mean (SD) | 9.8 (18.5) | 12.2 (18.1) | 13.7 (28.7) | 4.9 (9.9) |
| Median (Q1, Q3) | 2.0 (2.0, 3.2) | 2.0 (2.0, 12.0) | 2.0 (2.0, 6.5) | 2.0 (2.0, 3.0) |
| Min; Max | 1.0; 93.0 | 1.0; 50.0 | 1.0; 93.0 | 1.0; 44.0 |

Abbreviations: CI, confidence interval; NE, not estimable; Q, quartile; SD, standard deviation

^a^ Percentages based on patients at-risk.

^b^ Reverse Kaplan-Meier method

^c^ 95% confidence interval calculated using the log-log transformation.

^d^ CI not estimable due to heavy censoring

^e^ Counts ≤2 are masked (“–”) per institutional privacy policy; additional suppression applied to prevent back-calculation. Zero values are shown as reported.
